# Tumour Cell Size Control and Its Impact on Tumour Cell Function

**DOI:** 10.1111/cpr.70080

**Published:** 2025-06-17

**Authors:** Min Zhou, Mei Zhou, Yang Jin

**Affiliations:** ^1^ Department of Respiratory and Critical Care Medicine, Hubei Province Clinical Research Center for Major Respiratory Diseases, NHC Key Laboratory of Pulmonary Diseases Union Hospital, Tongji Medical College, Huazhong University of Science and Technology Wuhan China; ^2^ Key Laboratory of Biological Targeted Therapy Union Hospital, Tongji Medical College, Huazhong University of Science and Technology Wuhan China; ^3^ Hubei Province Engineering Research Center for Tumor‐Targeted Biochemotherapy Union Hospital, Tongji Medical College, Huazhong University of Science and Technology Wuhan China

**Keywords:** anti‐cancer immunity, cancer cell size, cancer metastasis, cancer stem cells, cell cycle, cell size, EMT, mTOR, tumour microenvironment

## Abstract

Cell size is an important component of cell morphological characteristics. It reflects the characteristics of the cell type, nutritional status, growth stage and physiological function. The cell size of cells of the same type tends to be homogeneous and stable. However, in tumour cells, mutations in cell cycle genes and cytoskeletal genes and overexpression of the corresponding signalling pathways often lead to large variations in tumour cell size. Tumour cells regulate cell size and growth and proliferation through multiple signalling pathways, such as PI3K/Akt/mTOR, Myc and Hippo pathways, which work together to regulate cell size and proliferation. This allows tumour cells to adapt to different survival environments. Alterations in cell size also cause tumours to perform different functions, leading to alterations in tumour stemness, invasive migration and anti‐tumour immunity by affecting immune cells in the tumour immune microenvironment. In this review, we describe the endogenous and exogenous factors affecting tumour cell size, analyse the mechanisms by which tumour cells regulate cell size and the effects of cell size on tumour malignancy and tumour immunity, summarise the potential therapeutic targets for cell size, and look forward to possible future research directions and clinical applications.

## Graphical abstract

The regulatory mechanism of the size of tumour cells and its impact on the functions of tumour cells, as well as a summary of potential therapeutic targets for the corresponding mechanisms.

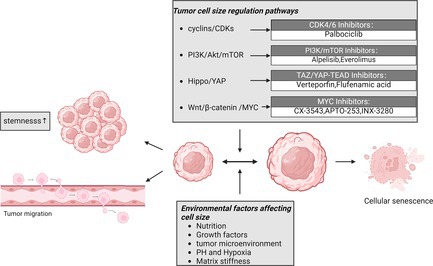

**Abbreviations**
4E‐BP1: Eukaryotic translation initiation factor 4E–binding protein 1
ACC: Acetyl‐CoA carboxylase
Akt: Abstract Protein kinase B
AMPK: Adenosine monophosphate–activated protein kinase
AP‐1: Activator protein–1
APC: Adenomatous polyposis coli
ARP2/3: Actin‐related protein 2/3
ASCT2: Alanine‐Serine‐Cysteine Transporter 2
Axin: Axis inhibition protein
BAD: BCL2–associated agonist of cell death
Bcl‐2: B‐cell lymphoma–2
Bcl‐xL: B‐cell lymphoma–extra large
bHLH‐ZIP: Basic Helix–Loop–Helix Leucine Zipper
CAFs: Cancer‐associated fibroblasts
CAK: CDK‐activating kinase
CDC25: Cell division cycle 25
CDK: Cyclin–dependent kinase
CECs: Corneal endothelial cells
CK1α: Casein Kinase 1 Alpha
CIP: CDK–interacting protein
COL1A1: Collagen Type I Alpha 1 Chain
CSC: Cancer stem cell
CSCs: Cancer stem cells
CSN8: COP9 Signalosome Subunit 8
Cdc42: Cell division control protein 42
CXCL16: C‐X‐C motif chemokine ligand 16
DCs: Dendritic cells
DHFR: Dihydrofolate reductase
DNMT1: DNA (cytosine‐5)‐methyltransferase 1
E2F: E2 promoter–binding factor
E2F3: E2F Transcription Factor 3
ECM: Extracellular matrix
EGF: Epidermal growth factor
EGFR: Epidermal Growth Factor Receptor
EMT: Epithelial–mesenchymal transition
EMT: Endothelial‐mesenchymal transition
ER: Oestrogen receptors
FACS: Fluorescence‐activated cell sorting
FA: Focal adhesions
FASN: Fatty acid synthase
FGF2: Fibroblast Growth Factor 2
FZD: Frizzled
GOT2: Glutamic‐Oxaloacetic Transaminase 2
GPCRs: G protein‐coupled receptors
GGF: Glial growth factor
GLUT1: Glucose transporters 1
GsMTx4: Grammostola spatulate mechanotoxin 4
GSK3β: Glycogen synthase kinase 3 beta
HGF: Hepatocyte growth factor
HGF: Hepatocyte Growth Factor
HER2: Human Epidermal growth factor Receptor 2
HIF‐1α: Hypoxia–inducible factor–1 alpha
HK2: Hexokinase 2
IGF‐1: Insulin‐like Growth Factor 1
IGFBP2: Insulin‐like growth factor‐binding protein 2
ISCs: Intestinal stem cells
IRF7: Interferon Regulatory Factor 7
JAK2: Janus kinase 2
KIP: Kinase–inhibitory protein
LATS1/2: Large tumour suppressor kinases 1/2
LC3: Microtubule–associated protein 1A/1B–light chain 3BNIP3
LCT: Large cell transformation
LEF: Lymphoid enhancer–binding factor
LKB1: Liver kinase B1
LRP5/6: Low–density lipoprotein receptor–related protein 5/6
LRP6: Low‐Density Lipoprotein Receptor‐Related Protein 6
MAPK: Mitogen–activated protein kinase
MAT1: Maturation–associated protein 1
Max: MYC‐Associated Factor X
MET: Epithelial morphology
MMPs: Matrix metalloproteinases
MMPs: Matrix metalloproteinases
MOB1A/B: Mps one binder kinase activator‐like 1A/B
MST1/2: Mammalian STE20‐like kinases 1/2
MYC: Myelocytomatosis oncogene
MyoD: Myogenic differentiation 1
NF‐κB: Nuclear Factor Kappa‐Light‐Chain‐Enhancer of Activated B Cells
NHE: Na+/H+ exchanger
NHE1: Na+/H+ exchanger 1
NKA: Na+/K+‐ATPase
NSCLC: Non–small cell lung cancer
Olig2: Oligodendrocyte Lineage Transcription Factor 2
Oct4: Octamer‐Binding Transcription Factor 4
OS: Overall survival
PARP1: Poly(ADP‐ribose) polymerase 1
PDGF: Platelet‐derived growth factor
PDK1: Pyruvate dehydrogenase kinase 1
PFS: Progression‐free survival
PFKFB3: 6–Phosphofructo–2–kinase/Fructose–2,6–bisphosphatase 3
PI: Phosphatidylinositol
PI3K: Phosphoinositide 3–kinase
PIP2: Phosphatidylinositol 4,5–bisphosphate
PIP3: Phosphatidylinositol 3,4,5–trisphosphate
PKB: Protein kinase B
PKB: Protein kinase B
PLCγ: Phospholipase C gamma
Pol I: RNA polymerase I
PTEN: Phosphatase and tensin homologue
RHEB: Ras–homologue enriched in brain
ROS: Reactive Oxygen Species
RTK: Receptor tyrosine kinase
RTKs: Receptor tyrosine kinases
Runx2: Runt–related transcription factor 2
Rb: Retinoblastoma protein
Rac1: Ras–related C3 botulinum toxin substrate 1
Ras: Rat sarcoma virus
SAV1: Salvador homologue 1
SCLC: Small cell lung cancer
SREBP: Sterol Regulatory Element–Binding Protein
SOD2: Superoxide Dismutase 2
STAT3: Signal transducer and activator of transcription 3
S6K: S6 kinase
TAZ: Transcriptional co–activator with PDZ–binding motif
TAMs: Tumour‐associated macrophages
TCF: T‐cell factor
TGF‐β: Transforming growth factor–beta
THZ1: 4‐(4‐Methylpiperazin‐1‐ylmethyl)‐N‐[5‐[(phenylthio)methyl]‐1,3‐thiazol‐2‐yl]benzamide
TK: Thymidine kinase
TKIs: Tyrosine kinase inhibitors
TLR9: Toll‐Like Receptor 9
TME: Tumour microenvironment
TNBC: Triple‐negative breast cancer
TNF: Tumour Necrosis Factor
TSC2: Tuberous sclerosis complex 2
TRPP2: Transient Receptor Potential Polycystin 2
TRPV4: Transient Receptor Potential Vanilloid 4
ULK1: Unc–51–like kinase 1
VEGF: Vascular endothelial growth factor
WGD: Whole‐genome doubling
Wnt: Wingless–related integration site
Wnt: Wingless–related integration site
YAP: Yes–associated protein

## 1 Introduction

Cell size typically refers to the volume of a cell. Additionally, cell mass, surface area‐to‐volume ratio, nucleus size, protein content and DNA‐to‐cell volume ratio are closely related to cell size and can therefore serve as indicators for assessing cellular dimensions [1, 2]. Cells in the human body with diverse functions and types exhibit characteristic sizes that reflect their physiological traits. Significant variations in size and morphology were observed among different cell types. For instance, red blood cells typically have a diameter of 6–9.5 μm, which is a compact size that enables them to navigate narrow capillary spaces and deliver oxygen to distal tissues. In contrast, the largest skeletal muscle fibres can extend up to 10s of centimetres in length, allowing them to generate substantial contractile forces required for limb movement and body locomotion [1, 3]. Size variations also exist among different types of tumour cells. Small cell lung cancer (SCLC) and non‐small cell lung cancer (NSCLC), both classified as lung tumours, exhibit distinct differences. SCLC, as a poorly differentiated tumour, features a higher nuclear‐to‐cytoplasmic ratio and rapid proliferation rate, resulting in a smaller cell size [1, 4]. In pathological diagnosis, SCLC is typically defined by tumour cells smaller than three times the average lymphocyte diameter (approximately 1.5 times the lymphocyte diameter, with lymphocytes averaging 7–15 μm), characterised by a nuclear‐to‐cytoplasmic ratio > 0.7 and a cell diameter generally ranging between 8 and 10 μm, while large cell tumours often exceed 30 μm in diameter, averaging 30–50 μm. Other small cell neuroendocrine carcinomas, such as those arising in the oesophagus, gastrointestinal tract, bladder and prostate, also demonstrate similarly small cell sizes and high aggressiveness. Hematologic malignancies like chronic lymphocytic leukaemia (CLL) and small lymphocytic lymphoma (SLL) exhibit tumour cells with scant cytoplasm and small diameters (6–10 μm). Burkitt lymphoma displays intermediate or relatively small cell sizes (10–25 μm in diameter) characterised by rapidly proliferating cells, and also exhibits a smaller cell size [5]; pleomorphic giant cell carcinoma is characterised by exceptionally large cells (30–100 μm in diameter) and is frequently observed in pancreatic, endometrial, non‐small cell lung, and bladder cancers. These cells often exhibit multinucleation, vigorous nuclear division and massive size, indicating a high degree of malignancy [6, 7, 8]. Notably, the giant cell variant of glioblastoma may feature tumour cells exceeding 400 μm in diameter and characterised by heightened glycolysis and reduced mitochondrial content, undergoes cytoplasmic dilution, leading to an increase in cell volume. Additionally, ion channels on the cell membrane regulate the influx and efflux of ions, such as K+ and Cl−, thereby influencing intracellular osmotic pressure, modulating tumour volume and facilitating tumour migration [9].

Tumour cells undergo dynamic changes in size at different growth and developmental stages. During the G1 phase of the cell cycle, cells absorb nutrients from their surroundings to support growth and development, preparing for mitosis by accumulating the necessary materials, synthesising RNA and ribosomes, and increasing their volume [10]. During G2/M transition, the Na+/H+ transporter NHE1/SLC9A1 is transiently activated, leading to an increase in intracellular hydrostatic pressure and a significant expansion in cell volume by up to 30%. This enlargement, accompanied by a decrease in cell density, facilitates the movement of chromosomes and organelles during mitosis [10, 11]. During mitosis, the degradation of focal adhesions (FA) reduces adhesion between the cell and extracellular matrix (ECM). The cell edges retract along the actin‐rich fibres, actin structures disassemble, and the dense cortex rich in actomyosin beneath the plasma membrane reorganises, rendering the cell morphology more rounded to better initiate mitosis [12]. During the late stages of mitosis, tumour cells often fail to undergo cytokinesis, potentially leading to endoreplication and whole‐genome doubling (WGD), resulting in polyploidy formation. This causes the nucleus of the tumour cell to increase in size, leading to overall morphological changes and significant heterogeneity in the daughter cells [13, 14]. Tumour cells undergo irreversible cell cycle arrest due to replication stress‐induced DNA damage, telomere shortening, or exposure to chemotherapeutic drugs, leading to cellular senescence. During this stage, mitochondria swell due to damage, and lysosomes increase within the cell, resulting in a significant enlargement of the cell volume and loss of polarity [2, 15, 16]. During the apoptotic phase, tumour cells undergo nuclear pyknosis, cytoplasmic dehydration, and condensation, leading to a gradual reduction in cell volume [17, 18]. When confronted with immune cell‐mediated killing or an altered microenvironment unsuitable for tumour proliferation, tumour cells enter reversible cell cycle arrest, a process known as cancer cell dormancy [19]. At this stage, dormant tumour cells exhibit cancer stem cell (CSC)‐like characteristics [20]. This type of cell undergoes morphological transformation through epithelial‐mesenchymal transition (EMT), exhibiting mesenchymal morphology as well as epithelial morphology (MET) and transitions between these two morphologies [21] to better traverse the vascular wall in cases of distant metastases [22]. Owing to reduced metabolism, decreased synthesis of substances, shrinkage of organelles, lowered activity of ion pumps and decreased intracellular water content, the volume of most tumour cells decreases during the dormant phase [23].

To facilitate the study of cell size, researchers typically use conventional optical or electron microscopy to roughly estimate the cell size; however, this method is unsuitable for large‐scale studies [24]. Combining the cellular information observed under the microscope with computer image analysis technology enables a more precise measurement of the cell size. This method first utilises traditional or electron microscopy to obtain high‐resolution cell images and then preprocesses these images using image processing techniques for enhanced accuracy. Finally, it employs computer analysis software to precisely measure the cell size based on features such as physical morphology or fluorescence staining. Additionally, flow cytometry can be used to analyse cell flow velocity and the intensity of cell membranes or intracellular fluorescent markers, thereby assessing cell size. After obtaining cell size information, to further investigate the functional characteristics of cells of different sizes, it is necessary to separate different cell populations. Using fluorescence‐activated cell sorting (FACS) technology, cells were categorised according to predefined size criteria for subsequent research and analysis [25]. Additionally, sieving analysis techniques can be employed to assess the distribution and proportion of cell sizes [26]. From the perspective of cell growth, methods that measure other indicators of cell size can indirectly indicate the cell size. For instance, cell growth rate can be calculated by measuring cell volume or dry mass and quantitative phase imaging techniques, such as Raman spectroscopy, can be used to measure intracellular protein synthesis rates [27, 28]; using the vibrating cantilever method, changes in the buoyancy and density of non‐adherent cells during the cell cycle can be measured [29, 30]. By fluorescently labeling the cell membrane, the displacement distance of fluorescence during cell growth can be measured, reflecting the growth amount [31]. Mass spectrometry‐based methods, such as amino acid isotope labeling in cells, are used to quantitatively analyse changes in protein content during cell growth [2]. Because the cell cycle is a dynamic process, cell size is significantly influenced by the cycle, and variations in size occur across different phases. Therefore, it is essential to observe the dynamic changes in the cell cycle to statistically analyse cell size across various periods. Live‐cell imaging microscopy, based on the fluorescent labeling of cell cycle genes, can be utilised to monitor cell cycle transitions and reflect the rate of cell division [32].

## 2 The Mechanisms of Tumour Cells in Regulating Cell Size

### 2.1 Mechanisms by Which Cells Sense Their Own Size

Cells can sense their size and regulate their growth rate and cycle progression accordingly, coordinating cell growth and division to control volume [33, 34]. There are three main theories on cell proliferation regulation: Sizer, Adder and Timer [35].

**FIGURE 1:**
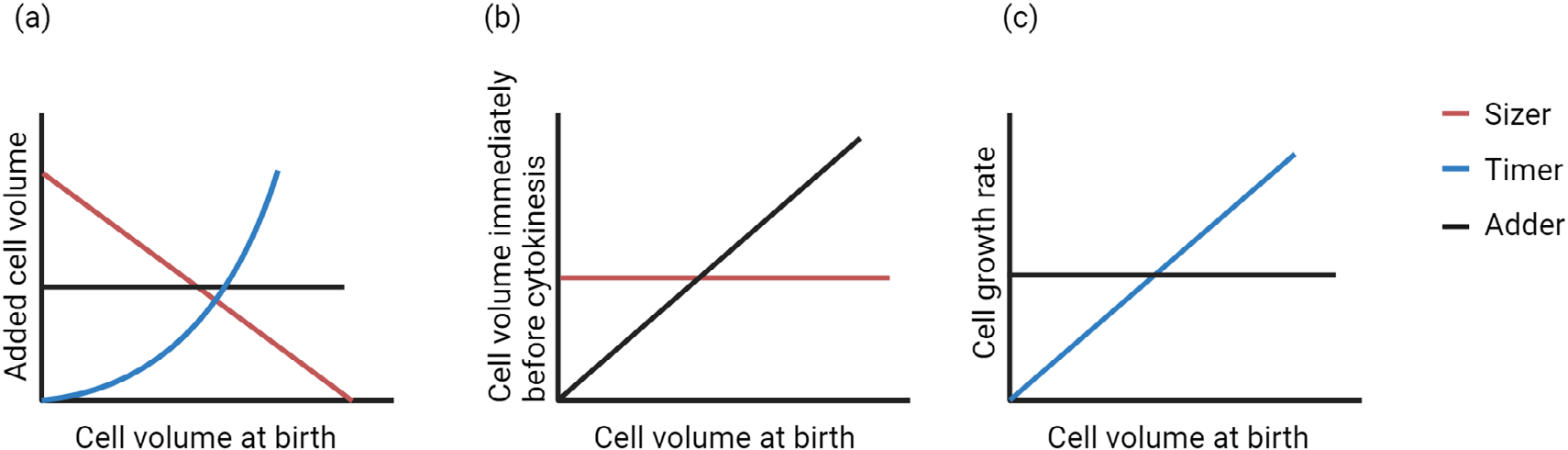
The influences of three theories on the growth volume and growth rate of cells. (a) In the Sizer theory, the increase in cell volume during the interphase of cell division is inversely proportional to the volume of the cell at birth; in the Timer theory, the increase in cell volume follows an exponential model relationship with the size at birth; in the Adder theory, the increase in cell volume is a constant value. (b) In the Adder theory, the volume of a cell before mitosis is directly proportional to its volume at birth; in the Sizer theory, the volume of a cell before mitosis is constant. (c) In the Timer theory, the cell growth rate is directly proportional to the volume of the cell at birth; in the Adder theory, the cell growth rate is constant.

**FIGURE 2:**
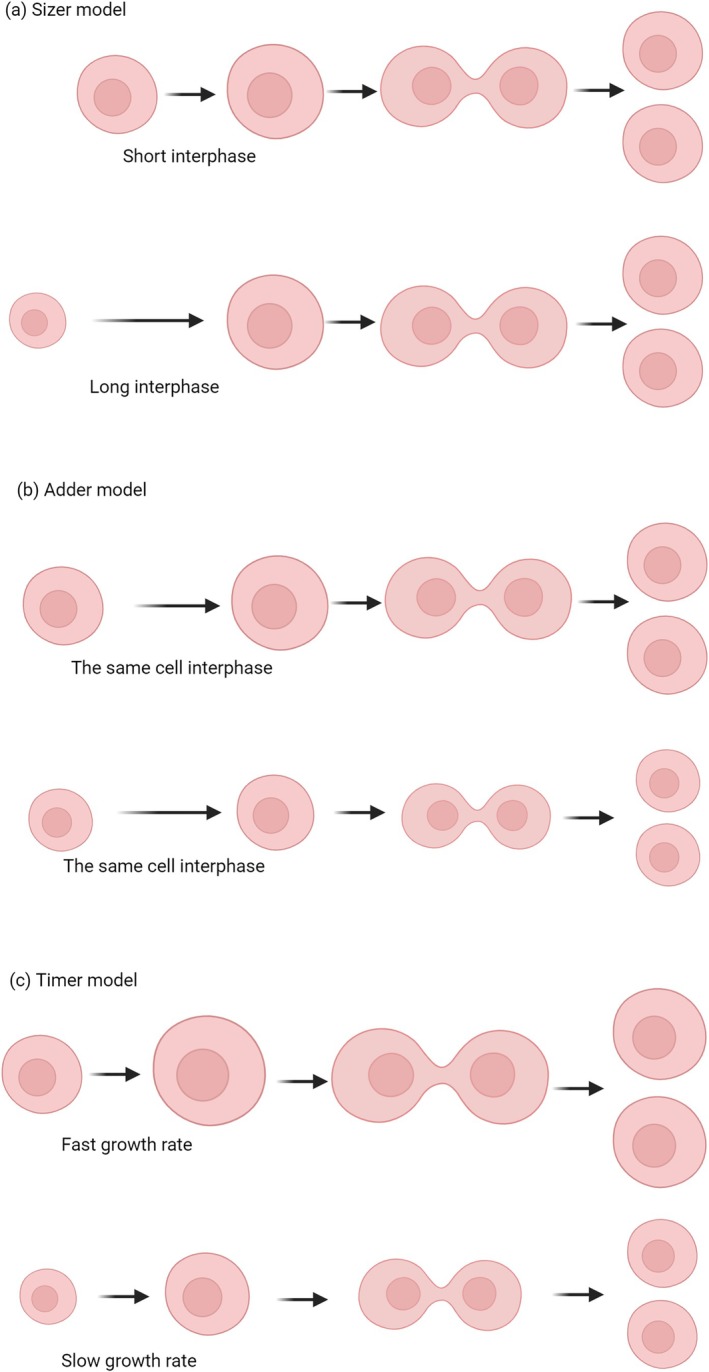
Three main theories on cell proliferation regulation. (a) In the Sizer theory, cells that are larger at birth have a smaller growth size before cell division, while cells that are smaller at birth need to grow to a larger size before they start to divide. (b) In the Adder theory, cells have a fixed growth amount in each cycle. Therefore, larger cells are also larger when they divide, and smaller cells are smaller when they divide. (c) In the Timer theory, cells grow according to an exponential model. The growth rate is proportional to the cell size. Larger cells grow faster, and the mass of cells that grow within the same period of time is greater for larger cells than for smaller cells.

The Sizer model posits that cells initiate division upon reaching a specific size. Consequently, cells that are larger at birth grow less before dividing, whereas cells that are smaller at birth need to grow more before they begin dividing. In other words, the timing of cell division is independent of the cell size at birth, but is triggered when the cell grows to a certain size [36]. According to this theory, the size of a cell at birth determines the timing of its proliferation. Since cells primarily regulate their size during the G1 phase, cells that are smaller at birth have a longer G1 phase, whereas cells that are larger at birth undergo mitosis more quickly [37]. The mechanism of this regulation may involve the activation of p38 in smaller cells to restrict division in cells below a certain size or the concentration of the cell cycle inhibitor RB decreases as cell volume increases during growth, thereby triggering mitosis [38]. Therefore, smaller cells need to grow in volume to reach the concentration required to initiate cell division [33] (Figures 1, 2).

The Adder model posits that cells exhibit a fixed amount of growth each cycle, meaning that regardless of their initial size, the growth during the interphase remains constant. As a result, larger cells remain larger at division, while smaller cells remain smaller at division. In this model, the volume of cell growth is regulated by the growth rate during the G1 phase, which is influenced by both cell type and growth conditions [39] (Figures 1, 2).

The Timer model assumes that cells initiate division after a specific growth period. If cells grow linearly, similar to the Adder model, both large and small cells divide after growing the same mass. If cells grow exponentially, the growth rate is related to the cell size. If the growth rate is proportional to the size, larger cells grow faster and accumulate more mass than smaller cells within the same time frame. Alternatively, if smaller cells grow faster, they accumulate more mass than larger cells simultaneously, resulting in cells of different sizes becoming similar in size before division [1, 40] (Figures 1, 2).

### 2.2 Tumour Cell Size Regulation Pathways

The regulation of cell size is closely tied to the cell cycle, particularly the G1 phase, as the coordination of growth and division timing by cells influences their size. When tumour cells grow to a certain volume, they initiate mitosis, and the mechanisms influencing the timing of division are related to certain cyclins, which are key regulators of cell size.

In the Cyclins‐related pathway, the Cyclin D‐CDK4/6 complex formed by the binding of Cyclin D and CDK4/6 phosphorylates the Rb protein, releasing the transcription factor E2F, which promotes the transition of the cell cycle from the G1 phase to the S phase. The binding of Cyclin E and CDK2 promotes the further phosphorylation of Rb, enabling the cell cycle to shift from the G1 phase to the S phase. The Cyclin B‐CDK1 complex promotes the transition of the cell from the G2 phase to the M phase.

In the PI3K/Akt/mTOR pathway, after the growth factor receptor on the cell membrane is activated, PI3K activates the phosphorylation of PIP2, generating the second messenger PIP3. PIP3 recruits the downstream molecule AKT to the cell membrane, where it binds to pyruvate dehydrogenase kinase 1 (PDK1), leading to phosphorylation and activation, and ultimately activates mTOR. Among them, PI3K is negatively regulated by PTEN. mTOR is involved in the formation of two complexes with different structures: mTORC1 and mTORC2. Once activated, mTORC1 contributes to the synthesis of components related to the cell membrane and organelle membranes, increases the cell's energy production, and reduces cellular autophagy.

In the Wnt/β‐catenin/MYC pathway, Wnt binds to the receptor Frizzled (FZD) and the coreceptor LRP5/6, inhibiting the degradation of β‐catenin and promoting β‐catenin to enter the nucleus and bind to TCF/LEF. This activates a series of downstream pathways, such as MYC, RAS‐MAPK and PI3K/AKT, thus regulating cell volume.

In the Hippo pathway, which is composed of core proteins such as mammalian STE20‐like kinases 1/2 (MST1/2), large tumour suppressor kinases 1/2 (LATS1/2), Salvador homologue 1 (SAV1), Mps one binder kinase activator‐like 1A/B (MOB1A/B), and so forth, when the Hippo pathway is inhibited, YAP/TAZ is activated, thereby promoting cell proliferation and affecting cell division.

#### 2.2.1 Cyclins Related Pathways

**FIGURE 3:**
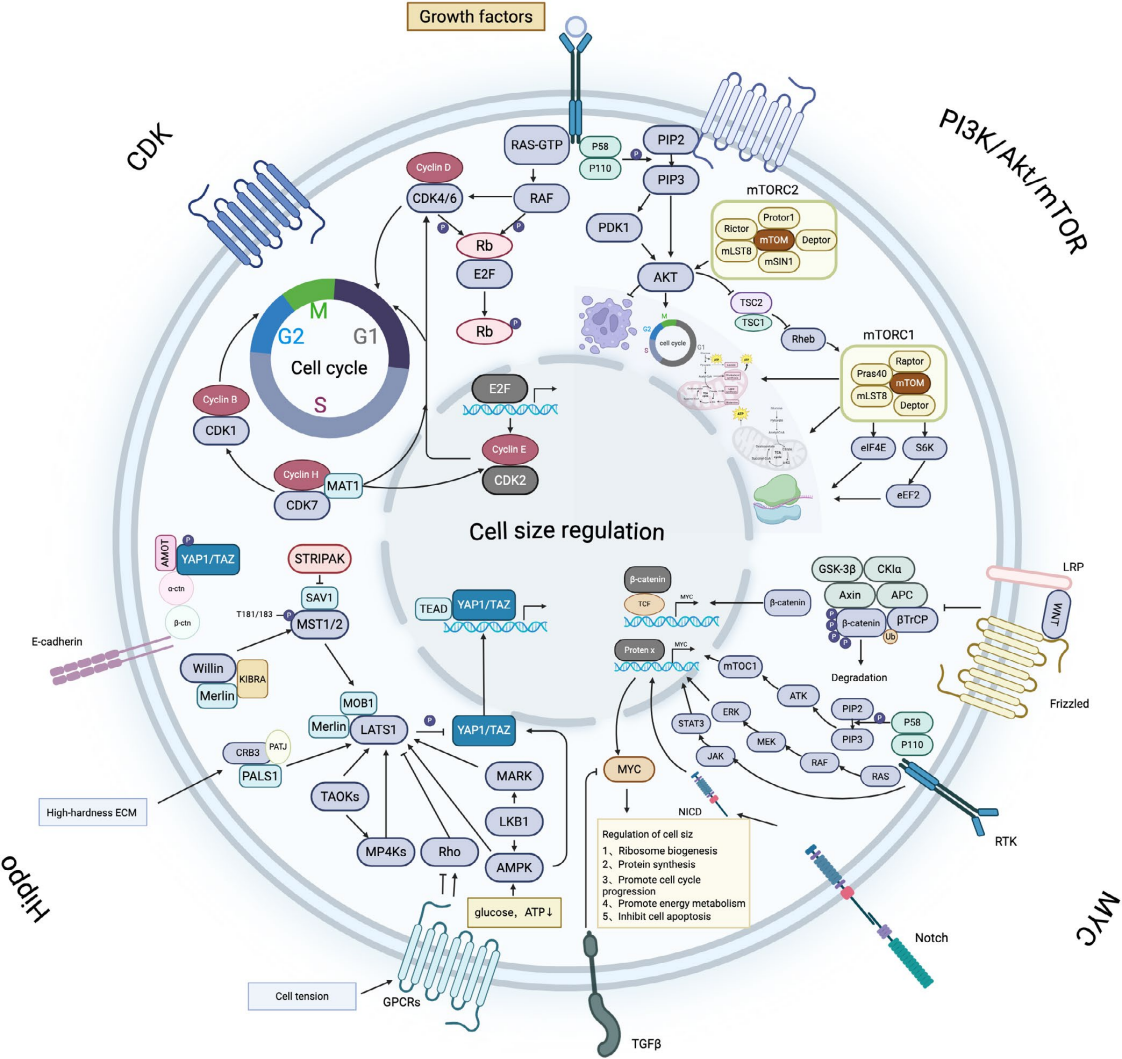
Tumour cell size regulation pathways.

Members of the cyclin family regulate various stages of the cell cycle by binding to cyclin‐dependent kinases(CDKs) [41]. They are regulated by signalling pathways, such as nutrients and growth factors, which maintain a balance between cell growth and cycle progression. Thus, when cell volume reaches a certain level, cyclins are activated to promote cycle progression. Inhibition of cyclin expression can halt the cycle and cause continuous cell volume expansion [42]. Cyclin D, a key regulator within this family, binds to CDK4/6. The resulting Cyclin D‐CDK4/6 complex phosphorylates the Rb protein, releasing transcription factor E2F and facilitating the transition from the G1 phase to the S phase of the cell cycle [43, 44, 45, 46] (Figure 3). Cyclin D is activated by the Ras‐MAPK or PI3K‐Akt pathways or by oncogenes such as Myc [47, 48]. It is overexpressed in various tumours, leading to unlimited proliferation, activation of metabolic pathways such as inhibition of TSC2 and activation of mTORC1, promoting substance synthesis [49]; and activation of transcription factors, such as HIF‐1α or Myc, promoting glycolysis to provide energy for tumour metabolism [50]. The accumulation of lactate within cells increases osmotic pressure, which causes fluid influx and cell swelling. The Cyclin D‐CDK4/6 complex can also indirectly phosphorylate ion channels or transporters through pathways such as PI3K/AKT, reducing ion transport function and altering osmotic pressure, leading to changes in cell volume [51]. Overexpression of Cyclin D inhibits signalling pathways, such as AMPK‐LKB1, and the activity of autophagy‐related factors and lysosome biogenesis‐related factors, such as TFEB, reducing the degradation of intracellular proteins and organelles, leading to their accumulation and an increase in cell volume [52, 53]. Cyclin E binds to CDK2 to further phosphorylate Rb, facilitating the transition of the cell cycle from the G1 phase to the S phase [54] (Figure 3). Excessive activation of Cyclin E triggers p53/p21, which inhibits cell cycle progression [55]. The Cyclin B‐CDK1 complex promotes the transition of cells from the G2 phase to the M phase [56] (Figure 3), phosphorylates and activates mitosis‐related proteins, such as the condensin complex and microtubule dynamics‐related proteins, thereby promoting chromosome condensation, spindle formation, and chromatid separation [57]. Cyclin B is regulated by kinases, such as Wee1, and phosphatases, such as CDC25 [58, 59], in order to prevent abnormal cell division due to unrepaired DNA damage or spindle attachment errors. In tumour cells, frequent overexpression of Cyclin B leads to heterogeneity in cell size and morphology.

#### 2.2.2 CDKs Related Pathways

Cyclin‐dependent kinases (CDKs) are crucial regulators of the cell cycle and gene transcription. The members of the CDK family include CDK1, CDK2, CDK3, CDK5, CDK7 and CDK9 [60]. Previous studies have shown that CDKs are inactivated in malignant tumours, leading to uncontrolled proliferation. Among the family members, CDK4/6 binds to Cyclin D to promote Rb phosphorylation, thereby releasing E2F and activating downstream pathways. This is one of the mechanisms by which CDK4/6‐mediated dysregulation of the tumour cell cycle results in uncontrolled tumour cell proliferation. This pathway is regulated by various factors that enable tumours to continue growing and proliferating in changing environments. For example, growth factors, such as EGF and PDGF, activate the RTK/Ras/MAPK pathway, and downstream molecules, such as MYC and AP‐1, promote the expression of Cyclin D [61, 62, 63]. Persistently activated PI3K/Akt/mTOR signalling in tumours reduces ubiquitination and degradation of Cyclin D, promotes synthesis and assembly of Cyclin D‐related proteins, and increases expression of the Cyclin D‐CDK4/6 complex [64]. In hepatocellular carcinoma, high expression of phosphoenolpyruvate carboxykinase 1 (PCK1) accelerates the transition of the cell cycle from the G1 phase to the S phase under low glucose conditions by activating the CDK/Rb/E2F pathway, promoting the growth and division of liver cancer cells [65]. In breast cancer cells, oestrogen binds to its receptor and directly activates the transcription of Cyclin D1, thereby promoting tumour proliferation [66]. Mutations in the tumour suppressor gene APC occur frequently in colorectal cancer. The loss of APC leads to activation of the Wnt/β‐catenin signalling pathway.β‐catenin binds to TCF/LEF, further activating the expression of Cyclin D1 and MYC [67]. The TGF‐β/Smad signalling pathway induces the expression of p15 and p21 [68] while simultaneously inhibiting the activity of MYC and Cyclin D. This regulatory mechanism modulates the expression of CDK4/6, contributing to the control of cell cycle progression. In pancreatic cancer, mutations frequently occur in the TGF‐β receptor [69], leading to the sustained activation of CDK4/6 in tumour cells [70]. This persistent activation drives the malignant proliferation of tumours. CDK1 binds to Cyclin B, facilitating the transition from the G2 phase to the M phase [71] (Figure 3). Inhibition of this molecule can induce cell cycle arrest in the G2 phase and lead to abnormal cell enlargement. CDK2 associates with Cyclin E and Cyclin A to promote the cell's entry into the S phase (Figure 3). This process is regulated and inhibited by the CIP/KIP family of proteins, such as p21, p27 and p57 [72]. CDK7 forms a CDK‐activating kinase (CAK) complex by associating with Cyclin H and MAT1. This complex regulates cell cycle progression by phosphorylating and activating other CDKs [73] (Figure 3).

#### 2.2.3 Rb Mediated Pathway

The cell cycle inhibitor retinoblastoma protein (Rb) is a tumour suppressor gene. Its concentration at cell birth was constant and did not increase with cell volume [1, 74]. During the G1 phase, as cell growth leads to an increase in volume, the concentration of this molecule is diluted, thereby weakening its inhibition of the cell cycle. When the concentration was diluted to a certain level, cell division was initiated [74]. When Rb is overexpressed, it prolongs the duration of the G1 phase and increases cell volume [74]. In various cancers, such as retinoblastoma, osteosarcoma [75], small cell lung cancer [76], breast cancer [77], and prostate cancer [78], the Rb gene often undergoes mutations leading to its inactivation, resulting in dysregulation of the tumour cell cycle.

Rb protein binds to and inhibits the E2F transcription factor. When upstream proliferative signals activate and phosphorylate Rb, it releases E2F, which then activates downstream cell cycle‐related genes, such as Cyclin E and CDK2 (Figure 3). Additionally, it upregulates the activity of RNA polymerase I/III [79], promoting the expression of nucleotide synthesis‐related genes, including DHFR and TK [80], thereby increasing tumour cell biosynthesis and facilitating tumour progression. In small cell lung cancer, due to the dysregulation of Rb, the functions of cell differentiation‐related factors, such as MyoD and Runx2, are inhibited [81], keeping the cells in a poorly differentiated state. Overactivation of mTORC1 signalling further drives continuous cell proliferation and division, resulting in reduced cell volume and a poorly differentiated phenotype in small cell lung cancer cells.

#### 2.2.4 PI3K/Akt/mTOR Pathway

Extensive previous research has demonstrated that the PI3K/Akt/mTOR pathway is a crucial component in regulating cell cycle and cell size‐related processes. When growth factor receptors on the cell membrane are activated, PI3K triggers the phosphorylation of PIP2, generating the second messenger PIP3. PIP3 then recruits the downstream molecule AKT to the cell membrane, where it binds to pyruvate dehydrogenase kinase 1 (PDK1), leading to its phosphorylation and activation. This cascade ultimately activates mTOR, which plays a pivotal role in modulating cell growth and apoptosis [82, 83] (Figure 3). There are multiple downstream molecules involved in regulating this pathway. Among them, PTEN negatively regulates PI3K by inhibiting the production of PIP3. As a tumour suppressor gene, PTEN plays an anti‐tumour role and is frequently found to be deficient or lost in various types of cancer [84]. Akt, also known as protein kinase B (PKB), is a serine/threonine kinase that is activated through phosphorylation by upstream regulators such as 3‐phosphoinositide‐dependent protein kinase (PDK), PI3K and receptor tyrosine kinases (RTKs) [85, 86]. Upon activation, Akt phosphorylates apoptosis‐related molecules to regulate cell survival. For instance, Akt phosphorylates BAD, one of the key pro‐apoptotic genes in the Bcl‐2 family. This phosphorylation inhibits BAD from binding to Bcl‐2 and Bcl‐xL to form heterodimers, thereby suppressing apoptosis [86]. In addition to inhibiting apoptosis, Akt can also mediate the phosphorylation of the tumour suppressor gene p21, thereby inhibiting cell cycle arrest and inducing cell proliferation [1, 85]. In the PI3K/Akt/mTOR pathway, mTOR is a downstream molecule of Akt and participates in the formation of two structurally distinct complexes: mTORC1 and mTORC2. Among these, mTORC1 is the target of the anti‐tumour drug rapamycin [87, 88]. mTORC1 is typically activated by nutrients and AKT‐induced inhibitory phosphorylation of TSC2 [89] (Figure 3). Nutrients, particularly the amino acids leucine, arginine and glutamine, are potent activators of mTORC1. These nutrients facilitate the translocation of mTORC1 to the lysosome, where it can interact with and be activated by RHEB. In the upstream pathway, AKT inhibits the formation of the TSC1‐TSC2 complex [90], which acts as a GTPase‐activating protein for GTP‐bound RHEB. When this complex is inhibited, mTORC1 is able to bind to GTP‐bound RHEB and become activated [91]. In cancer cells, mutations in the AKT or PI3K genes often lead to gene amplification, resulting in the overactivation of mTORC1, which promotes tumour cell proliferation [92]. In tumours, the abnormal expression of the PI3K/Akt/mTOR pathway activates downstream signalling, such as the phosphorylation of 4E‐BP1, leading to the release of the eukaryotic translation initiation factor eIF4E and promoting protein translation [93]. It also activates ribosomal S6 kinase (S6K), promoting ribosome biogenesis, increasing the synthesis of macromolecules such as proteins within the cell [94], and facilitating an increase in cell volume. mTORC1 is a critical regulator in the nutrient‐mediated control of cell growth. Under nutrient‐rich conditions, it activates SREBP, promoting the synthesis of cholesterol and fatty acids, which aids in the production of components necessary for cell and organelle membranes [95]. Additionally, it activates the expression of key glycolytic enzymes such as HK2 and PFKFB3, enhancing cellular energy production [96, 97]. Simultaneously, it phosphorylates ULK1, inhibiting the formation of autophagosomes and reducing the expression of autophagy‐related genes [98], such as LC3 and BNIP3. This decreases the degradation of cellular components, helping to maintain cell volume [99]. Akt activates members of the Rho GTPases family of cell signalling proteins, such as Rac1 and Cdc42 [100], which regulate the formation and arrangement of cytoskeletal proteins like actin within the cell. This leads to the generation of pseudopodia and induces morphological changes in the cell.

#### 2.2.5 Hippo Pathway

Dysregulation of the Hippo pathway is widely observed in various cancers, including lung cancer, colorectal cancer, ovarian cancer and liver cancer [101]. The Hippo pathway is composed of core proteins such as mammalian STE20‐like kinases 1/2 (MST1/2), large tumour suppressor kinases 1/2 (LATS1/2), Salvador homologue 1 (SAV1) and Mps one binder kinase activator‐like 1A/B (MOB1A/B). Its downstream signalling includes key regulators such as Yes‐associated protein 1 (YAP), transcriptional coactivator with PDZ‐binding motif (TAZ), and the transcriptional enhancer associate domain (TEAD) family, which collectively regulate physiological processes like cell proliferation and apoptosis and influence cell size [102]. (Figure 3) YAP/TAZ are central effector molecules in the Hippo pathway that can sense mechanical forces in the extracellular matrix [103] and modulate transcription accordingly. When the stiffness of the extracellular matrix increases, amplifying the mechanical forces acting on the cell, YAP/TAZ are activated, promoting cell volume expansion. Additionally, YAP can participate in regulating intracellular cytoplasmic pressure and cell size by modulating cytoskeletal tension, independent of cell division [104]. In cases of excessive cell proliferation, which alters mechanical forces between cells, LATS1/2 within the pathway is inhibited, leading to the dephosphorylation of YAP and TAZ. These molecules then translocate to the nucleus, where they bind to TEAD to drive cell proliferation [105]. When cells come into contact with each other, the Hippo pathway is activated to restrict cell size [106]. In various cancers, such as liver [107], breast [108] and lung cancer [109] the Hippo pathway is suppressed, resulting in the persistent activation of YAP/TAZ and uncontrolled tumour cell growth. Downstream targets of YAP/TAZ include cell cycle‐related genes like CCND1 and MYC, which accelerate cell cycle progression, and the activation of the mTORC1 signalling pathway, which promotes cellular biosynthesis and metabolism, thereby influencing cell size [110].

#### 2.2.6 Wnt/β‐Catenin/MYC Pathway

The Wnt family, including Wnt3a and Wnt1, interacts with the receptor Frizzled (FZD) and co‐receptors LRP5/6 to inhibit the phosphorylation and inactivation of β‐catenin by the degradation complex (APC/Axin/GSK3β/CK1α). The stabilised β‐catenin then translocates into the nucleus, where it binds to TCF/LEF to promote the transcription of downstream genes [111]. In colorectal cancer, one of the most frequently activated downstream molecules is MYC [112], a gene associated with cell proliferation. MYC is a proto‐oncogene encoding the bHLH‐ZIP transcription factor Myc, which plays a critical role in regulating cell size and proliferation [113]. The Myc family includes three genes: c‐Myc, N‐Myc and L‐Myc [114]. The expression of c‐Myc promotes the formation of cyclin (CCN) and cyclin‐dependent kinase (CDK) complexes, driving cell cycle progression and inducing uncontrolled cell proliferation [115].

The expression of Myc is regulated by multiple mechanisms. Myc binds to Max to regulate gene transcription, while six transcriptional repressors known as ‘Mxd proteins’ (Mxd1, Mxd2, Mxd3, Mxd4, Mnt and Mga) competitively bind to Max to modulate Myc activity [116]. Multiple pathways interact with MYC to coordinately regulate its expression. For example, growth factor‐activated RAS‐MAPK or PI3K/AKT pathways [117, 118] promote the expression of transcription factors such as AP‐1 and NF‐κB [119, 120], which activate the MYC promoter and enhance transcription (Figure 3). In T‐cell lymphoma, aberrant activation of Notch promotes MYC transcription [121]. In Burkitt lymphoma, chromosomal translocation t(8;14)(q24;q32) frequently leads to c‐MYC overexpression [122]. In colorectal cancer cells, mutations in the APC gene activate the Wnt/β‐catenin pathway, resulting in increased c‐MYC transcription [123]. Amplification of the 8q24 region, leading to elevated c‐MYC expression, is commonly observed in neuroblastoma [124], melanoma [125] and breast cancer [126].

In tumours, the overexpression of MYC activates RNA polymerase I (Pol I) and III (Pol III) and translation initiation factors such as eIF4E [127], enhancing protein synthesis and promoting metabolic reprogramming. MYC upregulates the expression of glucose transporters (GLUT1) [128], glutamine transporters (ASCT2) [129], fatty acid synthase (FASN) [130] and acetyl‐CoA carboxylase (ACC) [131], thereby promoting glycolysis and lipid synthesis. These changes provide the macromolecular building blocks necessary for tumour growth and proliferation. In liver or breast cancer, the overexpression of MYC leads to increased ribosome synthesis, prominent nucleoli and an accompanying increase in cell volume [132].

### 2.3 Environmental Factors Affecting Cell Size

#### 2.3.1 Nutrition

**FIGURE 4:**
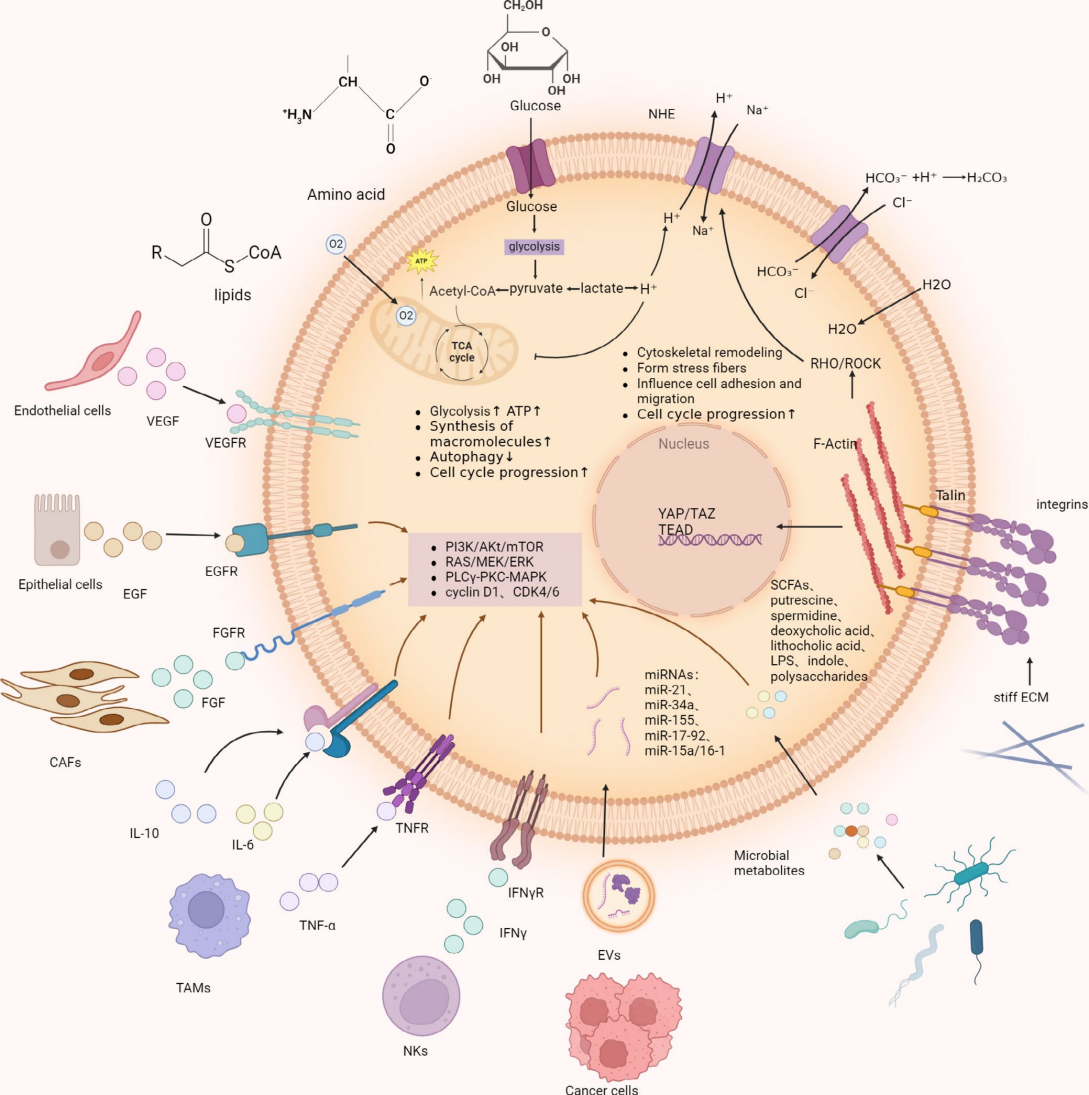
Environmental factors affecting cell size.

In the context of external stimuli influencing cell size, the availability of nutrients plays a significant role. Cell size is determined by the balance between the synthesis and degradation of intracellular macromolecules. When nutrients are abundant, the rate of macromolecule synthesis in cells is proportional to the concentration of nutrients in the environment, leading to larger cell growth [133]. The underlying mechanisms may involve nutrients influencing cell size by regulating cell cycle‐related signalling pathways. For example, in eukaryotes, mTOR kinases, including mTORC1 and mTORC2, along with their associated signalling pathways, are key regulators of nutrient utilisation and cell growth [134, 135] (Figure 4). As a result, cells grow to a larger volume before undergoing mitosis [136]. Tumour cells exploit the Warburg effect, utilising glycolysis to generate energy and accumulating large amounts of glycolytic intermediates such as lactate. This enables more efficient synthesis of nucleotides, lipids and amino acids, accelerating tumour proliferation. Tumour cells can uptake and utilise glucose at rates more than 10 times that of normal cells, leading to higher concentrations of intracellular nutrients and metabolites, and consequently, larger cell volumes [137].

Conversely, under nutrient‐deprived conditions, the rate of macromolecule synthesis decreases, and cells initiate division at a smaller size. This allows larger cells to undergo mitosis more rapidly, while smaller cells, due to slower growth, enter the division phase later [138]. In the tumour microenvironment, the demand for nutrients significantly increases due to the uncontrolled proliferation of tumour cells, resulting in relative nutrient scarcity. It is well known that tumour cells induce the expression of certain genes to reprogram their metabolism, enhancing nutrient utilisation efficiency. During this process, they undergo morphological and size changes to improve their adaptability to low‐nutrient conditions. For instance, when nutrient levels in the microenvironment decrease, mTORC1 and its associated signalling pathways are inhibited to coordinate cell growth and metabolism [139]. This reduces cell size and enhances autophagy in tumour cells, allowing the degradation and recycling of intracellular macromolecules to synthesise new materials, thereby coping with nutrient scarcity [140]. When glucose concentrations in the microenvironment drop, When glucose concentrations in the microenvironment drop, a cascade of signalling pathways is activated, triggering metabolic or functional adaptations in tumour cells. For example, researchers found that reducing glucose levels in the culture medium of HEK293 cells induced phosphorylation of p53‐Ser46, leading to tumour cell cycle arrest and apoptosis at the cellular level in one study. Therefore, this results in an increase in tumour cell volume and promotes apoptosis induced by cell cycle arrest [141, 142].

#### 2.3.2 Growth Factors

Cytokines regulate tumour cell proliferation through autocrine or paracrine mechanisms by binding to cell surface receptor tyrosine kinases (RTKs) or G protein‐coupled receptors (GPCRs), initiating downstream signalling pathways [143]. For example, EGF‐like growth factors and their receptor EGFR have been extensively studied in cancer. EGFR belongs to the ERBB family of cell surface receptor tyrosine kinases. When activated by ligand binding, it triggers a series of downstream signalling pathways, such as RAS–RAF–MEK–ERK–MAPK and PI3K–AKT–mTOR, upregulating integrin β1 and fibronectin to reorganise the cytoskeleton, alter hepatocyte morphology, and promote metastasis [144]. Vascular endothelial growth factor (VEGF), which is upregulated in various solid tumours such as lung and breast cancer, binds to RTKs and promotes the proliferation of tumour vascular endothelial cells, driving malignant progression. VEGFR, also a receptor tyrosine kinase expressed on vascular endothelial cells, activates the PLCγ‐PKC‐MAPK signalling pathway upon binding to VEGF [145].

Certain growth factors influence cell growth and size by controlling cell cycle progression. For instance, glial growth factor (GGF) accelerates Schwann cell cycle progression but does not promote cell enlargement. At higher concentrations of GGF, the cell cycle duration shortens, resulting in smaller cell size [146]. Certain cytokines exert effects on the cytoskeleton, thereby directly altering cell morphology and volume. For instance, fibroblast growth factor 2 (FGF‐2) stimulates corneal endothelial cells (CECs) to undergo endothelial‐mesenchymal transition (EMT) by activating phosphatidylinositol (PI) 3‐kinase and Ras‐related GTPases, reorganising the cytoskeleton, and inhibiting focal adhesion formation, causing cells to change from polygonal to spindle‐shaped [147]. In one study, researchers demonstrated that FGF2 is abnormally upregulated in the brains of animals lacking Rb family proteins, directly increasing cell size through the regulation of the FGF2 ligand gene by p107 and E2F3 [148]. The secretion of growth factors is frequently modulated by changes in the tumour microenvironment, when systemic nutritional conditions change. Endocrine hormones such as insulin promote the activation and expression of oncogenes, enabling tumours to adapt to changes in the internal environment and facilitating their progression. When insulin binds to its receptor, it initiates a series of phosphorylation events, including the activation of phosphoinositide 3‐kinase (PI3K), which cascades to activate downstream mTOR, thereby regulating cell size [148]. Hepatocyte growth factor (HGF), secreted by mesenchymal cells through paracrine action on target cells, plays a crucial role in organ regeneration and wound healing. In liver cancer, overexpression of the HGF/c‐Met pathway leads to excessive HGF secretion by mesenchymal cells, synergising with pathways such as PI3K/Akt/mTOR [149], MAPK/ERK [150], and Rho GTPases [151] to promote tumour proliferation, remodel the cytoskeleton, and facilitate metastasis. Giant cell‐type tumour cells are often observed in stem cell carcinomas with high c‐Met expression [152] (Figure 4).

#### 2.3.3 Tumour Microenvironment

The environment in which tumours reside is known as the tumour microenvironment (TME). Within this microenvironment, immune cells such as dendritic cells (DCs), macrophages, T cells, B cells, neutrophils and NK cells, along with the cytokines they secrete, collectively participate in tumour initiation, progression, and the shaping of tumour cell morphology [153]. These cells influence tumour phenotype and morphology through direct cell‐to‐cell contact and paracrine signalling. Cancer‐associated fibroblasts (CAFs) secrete growth factors such as HGF, IGF‐1, FGF and TGF‐β, activating pathways like PI3K/Akt/mTOR and MAPK, which promote tumour proliferation, increase macromolecule synthesis, and lead to cell volume expansion [154]. CAFs also secrete collagen and matrix metalloproteinases (MMPs). Researchers validated the increased secretion of MMP11 by cancer‐associated fibroblasts (CAFs) in the PDAC microenvironment at both tissue and cellular levels. In a separate study, it was demonstrated that anaplastic thyroid carcinoma (ATC) cells upregulate CREB3L1 expression, which activates α‐SMA‐positive CAFs to enhance fibrillar collagen secretion. The factors secreted by CAFs enhance the mechanical forces exerted by the extracellular matrix on cells, remodelling the cytoskeleton and increasing cell volume to better adapt to the environment [155] (Figure 4).

Tumour‐associated macrophages (TAMs), particularly the M2 subtype, play a significant role in promoting malignant tumour progression. TAMs secrete cytokines such as IL‐6, IL‐10 and TNF‐α, activating the STAT3 and NF‐κB pathways to accelerate cell cycle progression [156]. Endothelial cells secrete VEGF and FGF, promoting tumour angiogenesis and providing oxygen and nutrients to support tumour growth and volume expansion. In one study, breast cancer cells metastasising to the liver were found to increase the number of NK cells in the liver, upregulate IFNγ expression, and maintain tumour cells in a dormant state with smaller cell volumes [157].

The tumour microenvironment also contains extracellular vesicles carrying growth factors, miRNAs regulating cell proliferation‐related genes, and proteins involved in cytoskeletal remodelling. These vesicles act through autocrine or paracrine mechanisms to bind growth factors to their receptors and activate corresponding pathways (Figure 4). For example, breast cancer cells secrete HER2‐carrying exosomes [158] that activate downstream mTORC1, increasing ribosome synthesis, macromolecule translation, and cell volume. Colorectal cancer cell‐derived exosomes carrying miR‐21 inhibit PTEN [159], thereby activating the Akt/mTOR signalling pathway and enhancing tumour cell glycolysis and protein synthesis.

Microorganisms within tumours are a current research hotspot, as they can influence tumour phenotype and drive tumour progression. For instance, one study comparing cells infected with Fusobacterium and Porphyromonas to uninfected cells found upregulation of hypoxia‐related pathways such as the TNF pathway, activation of growth and proliferation‐related pathways like PI3K/Akt/mTOR and MAPK, and promotion of epithelial‐mesenchymal transition (EMT) and other phenotypic changes [160]. In colorectal cancer, the FadA adhesin secreted by *Fusobacterium nucleatum* binds to E‐cadherin on tumour cells, activating the β‐catenin pathway and promoting tumour proliferation [161]. In cervical cancer, HPV nucleic acids bind to TLR9, activating IRF7 and NF‐κB, and accelerating cell cycle progression [162].

#### 2.3.4 PH and Hypoxia

Tumour cells exhibit heightened metabolic activity, utilising the Warburg effect to undergo glycolysis, which results in the accumulation of large amounts of lactate within the cells [163], leading to a decrease in intracellular pH. The increase in hydrogen ion concentration induces the activation of the Na^+^/H^+^ exchanger (NHE), causing a significant influx of sodium ions. Concurrently, the compensatory decrease in HCO_3_^−^ concentration due to elevated H^+^ levels leads to the efflux of intracellular HCO_3_^−^ through the Cl^−^/HCO_3_^−^ exchanger, resulting in the accumulation of Cl^−^ within the cell (Figure 4). This increases intracellular osmotic pressure, causing fluid influx and cell swelling. When the pH drops too low, it affects mitochondrial activity, reducing energy production and inhibiting the activity of cyclin D1, CDK4/6, and mTORC1, weakening cellular synthesis and causing cell cycle arrest. The decrease in intracellular Ca^2+^ concentration affects actin polymerisation, disrupting cytoskeletal maintenance of cell morphology and reducing cell volume. For instance, when extracellular pH decreases, the activity of integrin α2β1 in human melanoma MV3 cells is inhibited, leading to a significant reduction in cell migration capacity. This study further revealed that extracellular pH and NHE (sodium‐hydrogen exchanger) activity critically influence cellular morphology. Specifically, cells exhibit distinct morphological changes under different conditions: Dendritic morphology at pH 6.6, amoeboid shape at pH 7.0, and a spherical phenotype at pH 8.1 in the presence of NHE inhibitors [164]. Elevated H^+^ concentration induces autophagy, activating the AMPK/mTOR pathway, which allows cells to maintain nutrient supply by degrading organelles and cytoplasm, leading to a reduction in cell volume.

Prolonged exposure to an acidic environment induces phenotypic changes in tumour cells to adapt to these conditions. For example, it drives the expression of stemness markers such as Olig2, Oct4 and Nanog, increasing the proportion of cancer stem cells (CSCs). It also promotes angiogenesis and enhances the invasive and migratory capabilities of tumour cells [165]. Hypoxia in the tumour microenvironment further influences tumour cell characteristics. Due to uncontrolled tumour cell proliferation and enhanced oxidative phosphorylation, oxygen is excessively consumed, leading to hypoxia in the tumour microenvironment. Under these metabolically suppressed conditions, tumour cells regulate the expression of genes such as HIF1‐α, NF‐κB, and Notch transcription factors to undergo epithelial–mesenchymal transition (EMT). This reduces adhesion to the extracellular matrix and between cells, transforming cells from an epithelial to a mesenchymal phenotype, making it easier for them to detach from the primary site and metastasize to more favourable locations via the bloodstream. One study confirmed that CSN8 is significantly upregulated in colorectal cancer tissues and is associated with poor prognosis, likely through the activation of the HIF‐1α signalling pathway, inducing EMT and tumour cell dormancy, thereby helping tumours adapt to hypoxic conditions and promoting cancer metastasis [166].

#### 2.3.5 Matrix Stiffness

In the tumour microenvironment, most tumour cells can sense various mechanical forces through mechanosensors and respond to them via mechanotransduction processes, including changes in cell morphology [167]. When the stiffness of the extracellular matrix (ECM) increases, cells sense external mechanical forces through integrins and activate the Rho/ROCK pathway, promoting the formation of actin stress fibres and increasing cell volume [168] (Figure 4). In pancreatic ductal adenocarcinoma, the ECM contains abundant collagen fibres, leading to extensive fibrosis and increased stiffness, which activates ROCK‐mediated cell volume expansion [169]. External mechanical forces, such as compression, cause nuclear pores to allow more small molecules, including YAP, to pass through. YAP, a transcriptional regulator modulated by the Hippo pathway, undergoes nuclear translocation in response to mechanical forces, where it binds to TEAD in the nucleus, driving cell proliferation and promoting tumour growth [170]. (Figure 4). Another critical molecule involved in this process is TAZ, which is a transcriptional coactivator and downstream effector of the Hippo pathway, works synergistically with YAP to regulate cytoskeletal tension and influence cell cycle progression, thereby modulating cell size [171]. In breast cancer, fibrosis increases tumour stiffness, activating YAP/TAZ and leading to cell volume expansion and pseudopod formation [172]. In melanoma, mechanical forces exerted by the surrounding environment activate YAP, promoting negative durotaxis [173], which reduces melanoma cell volume and facilitates migration to areas of lower stiffness [174]. During zebrafish heart valve morphogenesis, mechanosensitive ion channels such as Piezo1, TRPP2, and TRPV4 on the cell membrane sense mechanical forces generated by ECM components like hyaluronic acid, regulating cell size [175]. Mechanical forces can also activate the Wnt/β‐catenin signalling pathway to modulate cell growth. For example, in a hyperosmotic environment, mechanical compression on intestinal epithelial cells upregulates LRP6 expression, activating the Wnt/β‐catenin pathway and causing intestinal stem cells (ISCs) to grow larger and enhance their stemness [176].

External environmental stimuli can affect the volume of tumour cells. When nutrients such as glucose, lipids, and amino acids are abundant, the synthesis of tumour substances and the production of energy are accelerated, leading to an increase in cell volume. Growth factors such as EGF, VEGF, FGF, IL‐10, IL‐6, TNF‐α, IFNγ produced by TAMs and NKs and miRNAs carried in tumour‐derived EVs promote tumour proliferation by activating pathways such as RAS–RAF–MEK–ERK–MAPK and PI3K–AKT–mTOR. Excessive lactic acid produced by tumour cells leads to an increase in hydrogen ion concentration, which promotes sodium–hydrogen exchange, increases the intracellular concentration of sodium and chloride ions, increases osmotic pressure, and causes cell swelling. The stiff extracellular matrix activates the Rho/ROCK pathway through integrins, promoting the formation of actin stress fibres and increasing cell volume.

## 3 The Influence of Tumour Cell Size on Cell Function

### 3.1 Cell Size Influences the Synthesis and Metabolism of Substances in Tumours

**FIGURE 5:**
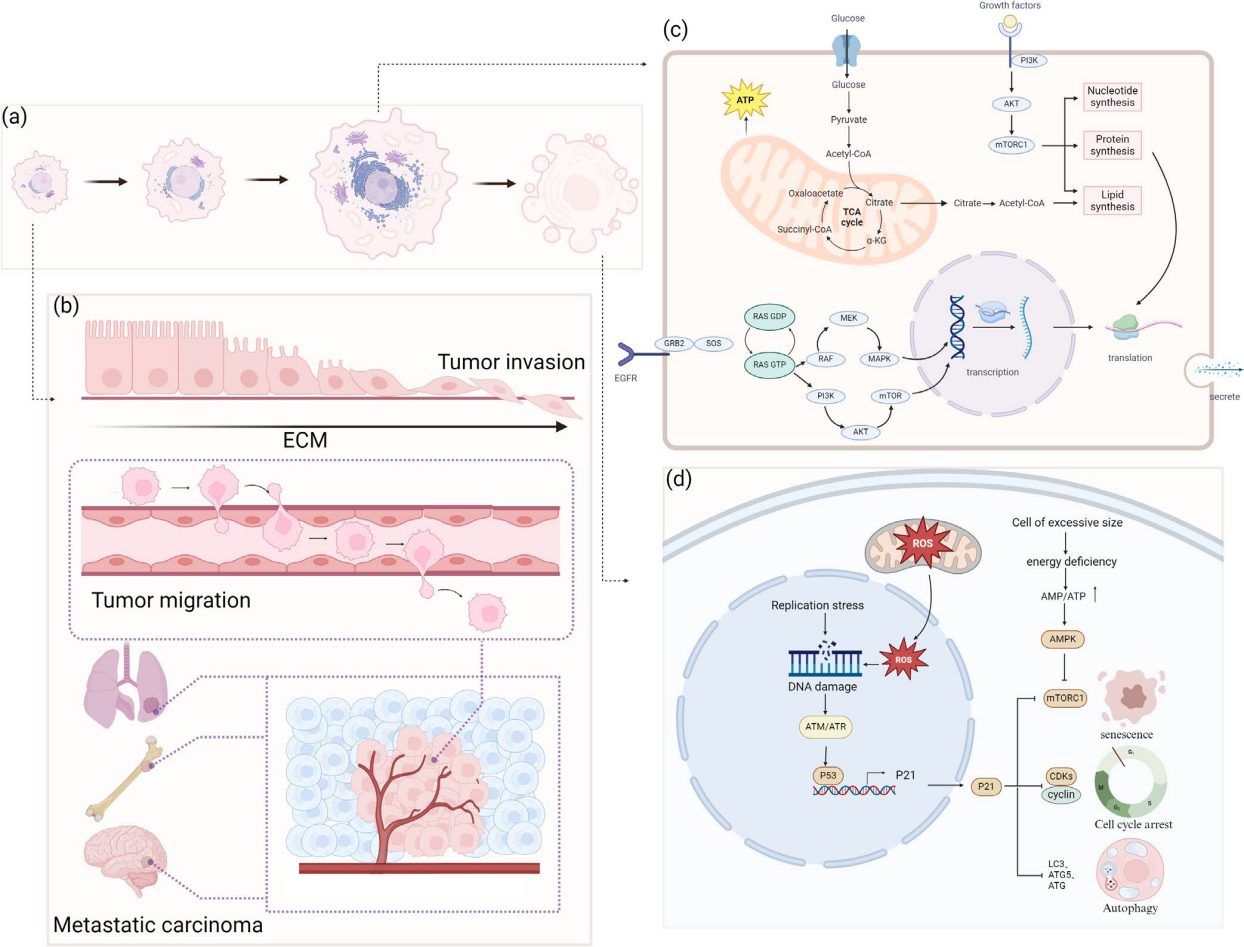
The influence of tumour cell size on cell function. (a) The process of tumour cells progressing from small to large in size and ultimately undergoing senescence. (b) Tumour cells undergo epithelial–mesenchymal transition (EMT) through interactions with the extracellular matrix (ECM), enabling them to invade the basement membrane, intravasate into the bloodstream, and disseminate to distant organs, thereby facilitating tumour metastasis. (c) In larger tumour cells, mitochondria provide an increased energy supply to support anabolic metabolism, while the activation of the RAS–RAF‐MAPK and PI3K‐Akt signalling pathways enhances gene transcription and translation, thereby augmenting the synthesis of proteins, lipids and nucleic acids. (d) When cells undergo excessive enlargement, the accumulation of reactive oxygen species (ROS) in mitochondria leads to mitochondrial dysfunction. Excessive free radicals damage DNA, activate AMPK and upregulate p21, resulting in cell cycle arrest and the induction of cellular senescence.

Cell size and morphology are closely linked to cellular physiological functions [177], particularly biosynthetic capacity. In larger cells, the burden of anabolic metabolism increases, prompting cells to augment mitochondrial numbers to meet the heightened physiological demands associated with increased cell size, thereby providing more energy for cellular synthesis and metabolism [178]. The demand for lipids, proteins and other components essential for maintaining cellular physiological activities also rises, leading to increased transcription and translation of genes, including higher quantities of polymerases themselves [179, 180]. This enhances tumour metabolism, macromolecule synthesis and secretion, thereby influencing tumour proliferation, invasion, migration and interactions with cells in the microenvironment. In lung adenocarcinoma, proliferating cells increase organelle numbers and RNA transcription to boost macromolecule synthesis and energy supply, resulting in generally larger cell volumes [181] (Figure 5a). However, when cell volume becomes excessively large, the number and energy production of mitochondria may not match the increased energy demands, potentially inducing apoptosis [182]. Although mitochondrial abundance correlates with cell size, mitochondrial membrane potential and oxidative phosphorylation are highest at intermediate cell sizes. Therefore, to maximise energy production, cell volume is often maintained within a specific optimal range [183]. Excessively large cells upregulate substances like SOD2 to neutralise superoxides, but excessive ROS accumulation in mitochondria can impair their function [184]. Excessive cell enlargement can also dilute ribosome concentration within the cell, affecting protein synthesis and disrupting normal physiological functions, ultimately inducing apoptosis [15].

Since the rates and directions of various biochemical reactions within cells are determined by the concentrations of reactants, biochemical reactions differ in cells of varying sizes. Additionally, cell size directly influences the surface area‐to‐volume ratio and the spatial distance of molecular transport within the cell [185]. Biochemical reaction rates are optimal for cell survival only when cell volume varies within a certain range. Beyond this range, membrane transport efficiency is affected, impairing cellular anabolic and metabolic processes [186]. In summary, cellular synthesis and metabolism are positively correlated with cell volume up to a certain point, beyond which energy production and synthesis efficiency decline, potentially inducing cellular senescence and apoptosis (Figure 5d).

### 3.2 The Relationship Between Cell Size and Tumour Stemness

Stem cells possess the ability to self‐renew and differentiate into cells with diverse functional and morphological characteristics. Many studies have found that one of the most common features of stem cells is their small size, often being non‐adherent and growing in clusters [187]. Cancer stem cells (CSCs) within tumour tissues are similar to stem cells in normal tissues, representing a subset of cells with unlimited proliferative potential and the ability to differentiate [188]. These cells typically undergo asymmetric division during proliferation, preserving their stemness while generating differentiated progeny [189]. The stemness of tumour stem cells is strongly correlated with cell size. For example, in breast cancer, smaller cells express higher levels of stemness markers such as OCT4, NANOG and SOX2. Researchers have enriched hTERT‐GFP(+) MCF7 breast adenocarcinoma cells based on telomerase transcriptional activity, identifying a population with strong stemness characteristics. Similarly, sorting cells by size yielded a population with corresponding stemness features [190], indicating a strong correlation between cell size and tumour stemness. In another study, researchers sorted fully undifferentiated prostate cancer cells (PC3) into large and small populations using flow cytometry and subcutaneously implanted them into mice. The results revealed that smaller cells were more tumorigenic, forming larger and more numerous tumours [191]. In contrast, in leukaemia, larger tumour cells exhibit stronger stemness.

### 3.3 The Impact of Cell Size on Tumour Invasion and Metastasis

To enter the bloodstream or lymphatic system for distant metastasis, tumour cells must traverse the basement membrane and vascular endothelial cells, often undergoing morphological and phenotypic changes to better deform and squeeze through narrow gaps into the circulation. The most common phenotypic change is the epithelial‐mesenchymal transition (EMT), during which cells undergo significant morphological alterations, becoming elongated and spindle‐shaped, with reduced surface folds [192] and loss of adhesive junctions and apical‐basal polarity (Figure 5b). Smaller cells are more deformable, allowing them to pass through the basement membrane and endothelium more easily, thus exhibiting higher invasiveness [193, 194]. For example, in melanoma, smaller cell subpopulations demonstrate stronger penetration capabilities. Due to their smaller surface area, smaller cells have less stable focal adhesion distribution, facilitating rapid migration, while larger cells are better suited for slow, sustained migration.

Therefore, tumour cells that disseminate from the primary site into the bloodstream often exhibit a highly plastic and compact morphology, enabling their efficient migration through tissues. For example, circulating tumour cells (CTCs) exhibit high heterogeneity, with various morphologies including epithelial, epithelial‐mesenchymal and mesenchymal phenotypes. Notably, smaller CTCs are more likely to migrate and colonise distant organs. Consequently, abnormal morphological changes in CTCs are strongly correlated with poor prognosis in cancer patients [193]. In a recent study, a research team enrolled 48 eligible non‐small cell lung cancer (NSCLC) patients and analysed pre‐treatment peripheral blood samples to investigate the correlation between CTC features and clinical outcomes. The study revealed that patients with smaller CTCs exhibited a higher incidence of advanced lymph node metastasis and shorter progression‐free survival (PFS), indicating a poorer prognosis [195]. In certain haematological malignancies, larger cells often exhibit stronger invasiveness. For instance, in T‐cell lymphoma, aberrant PEG10 expression increases cell size, promotes cell proliferation and leads to large cell transformation (LCT) in advanced stages, enhancing tumour aggressiveness [196]. Enlarged cells remodel the tumour microenvironment by secreting more collagen (COL1A1) and matrix metalloproteinases (MMPs), facilitating tumour invasion and migration. For example, in colorectal cancer, increased cell size positively correlates with high expression of MMP2/9, promoting tumour metastasis [197].

### 3.4 The Association Between Cell Size and Senescence Induction

Due to rapid proliferation, tumour cells undergo continuous division, leading to telomere shortening and DNA damage after repeated DNA replication. This activates the p53‐p21 pathway, inducing cell cycle arrest. However, cells continue to grow and increase in size. As cell volume expands, metabolic demands rise and mitochondria produce more reactive oxygen species (ROS), further damaging DNA. The synthesis of macromolecules such as proteins cannot keep up with the increasing cell volume, diluting the cytoplasm and reducing DNA abundance, slowing cell division and ultimately inducing senescence [15] (Figure 5d). Therefore, cell enlargement is strongly associated with senescence. Overactivation of oncogenes such as RAS, BRAF and MYC or suppression of tumour suppressor genes like PTEN, as well as DNA damage, intracellular hypoxia and oxidative stress, can activate the P53/P21 pathway, inhibit CDKs, and lead to cell cycle arrest, also inducing senescence. For example, in liver cancer, abnormal proliferation‐induced cell enlargement activates the p21 gene, driving cells into a senescent state [198] (Figure 5d). Cancer cells with DNA damage often exhibit significant volume increases, sometimes up to four times their original size [199]. When DNA damage is combined with mTOR inhibition, protein synthesis and metabolism are suppressed, halting cell enlargement and reducing the expression of senescence‐associated markers, indicating that inhibiting cell enlargement can mitigate senescence [200, 201]. Nectin‐4 is often highly expressed in senescent cells, and its overexpression typically leads to increased cell volume. Knocking down Nectin‐4 suppresses cell enlargement caused by senescence without affecting senescence itself [202]. Senescent vascular endothelial cells are generally larger than younger ones. Restricting excessive endothelial cell enlargement, such as by inhibiting CDC42 activity and stimulating YAP1 nuclear translocation, can promote cell proliferation and inhibit senescence [203].

## 4 Guidance for Cancer Treatment

### 4.1 Targeting the Cell Cycle

Excessive cell size can reduce ribosome and protein abundance, thereby affecting cell cycle progression. Therefore, inducing cell cycle arrest using cell cycle inhibitors and allowing continued cell growth to increase volume, leading to premature senescence, offers a novel approach for cancer treatment [204]. Targets in the cell cycle that are commonly addressed in therapeutic strategies include CDK4/6, CDK7, Cyclin D1, PLK1, Wee1, Aurora A/B and CDK1 [205]. By halting cells at a specific phase, tumour growth can be controlled. Among these, CDK4/6 inhibitors have been extensively studied. FDA‐approved CDK4/6 inhibitors, such as palbociclib, ribociclib and abemaciclib, are primarily used in breast cancer treatment [206]. Recent studies increasingly highlight the therapeutic potential of cycle inhibition in various cancers. CDK7 inhibitors induce cell cycle arrest and transcriptional suppression, particularly affecting super‐enhancer‐associated genes in cancer, and show potential in overcoming tumour resistance. Additionally, cancer cells are more sensitive to CDK7 inhibitors than normal cells, minimising DNA damage and side effects in healthy tissues [207]. Cyclin‐dependent kinase 3 (CDKL3) is a cell cycle regulator that directly phosphorylates Rb and CDK4, stabilising CDK4 and promoting cell cycle progression, thereby driving tumour growth and proliferation. The CDKL3 inhibitor HZ1 effectively blocks tumour cell cycle progression and addresses resistance to CDK4/6 inhibitors [208]. The small molecule inhibitor DDO‐5936 inhibits the protein interaction between Hsp90 and Cdc37, suppressing colorectal cancer cell cycle progression [209]. BLU‐222, a CDK2 inhibitor, has shown promising efficacy in clinical trials for ovarian cancer [210]. In acute myeloid leukaemia (AML), targeting PLK1 with Volasertib inhibits spindle formation, preventing cell division [211].

Antitumor drugs targeting the cell cycle to arrest it in the G2 phase, thereby inhibiting DNA replication caused by vigorous tumour cell division, typically include platinum‐based agents such as carboplatin and cisplatin; topoisomerase inhibitors like etoposide and doxorubicin; and microtubule‐targeting agents such as paclitaxel that disrupt mitosis. Rapidly proliferating tumour cells, characterised by accelerated cell cycle progression and smaller cell volume due to limited growth during the G1 phase, tend to exhibit heightened sensitivity to cell cycle‐inhibiting antitumor drugs. SCLC represents such a cell type, with its first‐line therapy involving etoposide combined with platinum‐based agents targeting its rapid proliferation. In contrast, LCLC, which demonstrates lower proliferative activity than SCLC, shows reduced sensitivity to these chemotherapeutic drugs. A cohort study revealed that SCLC patients receiving adjuvant chemotherapy with cisplatin and etoposide had significantly improved prognosis, whereas for pulmonary large cell neuroendocrine carcinoma, no significant association was observed between adjuvant chemotherapy administration and prognosis [212].

### 4.2 Targeting the Cell Growth Signalling Pathway

In tumours, pathways related to proliferation and metabolism, such as mTOR, PI3K/AKT and MYC, are often abnormally activated and overexpressed. Under the influence of their downstream signalling, tumour cells increase macromolecule synthesis, leading to growth and volume expansion. Targeting growth factor receptors and their downstream signalling pathways can effectively inhibit tumour proliferation. For example, in non‐small cell lung cancer (NSCLC), EGFR overexpression is common, and EGFR inhibitors like gefitinib have been clinically effective in suppressing tumour growth [213]. In melanoma, targeting the BRAF V600E mutation with inhibitors such as vemurafenib has shown significant therapeutic benefits [214]. In HER2‐positive breast cancer, HER2 overexpression is targeted by trastuzumab, which is widely used in clinical practice [215]. Inhibitors targeting the PI3K/AKT/mTOR pathway, such as everolimus and alpelisib, are used to treat various cancers, including renal cell carcinoma [216] and breast cancer [217], and have demonstrated efficacy in clinical trials. However, monotherapy with targeted agents often leads to drug resistance in tumour cells. Combining inhibitors targeting the same pathway or using them alongside chemotherapy drugs can effectively overcome resistance and improve treatment outcomes.

### 4.3 Targeting Metabolic Reprogramming

Due to their heightened metabolic activity, tumour cells exhibit increased synthesis of macromolecules such as proteins, lipids, and nucleotides, leading to cell volume expansion. Glutamine plays a critical role as an intermediate in the synthesis of proteins and nucleotides in tumours. The glutaminase inhibitor CB‐839 has shown promising efficacy in treating certain high‐metabolism tumours. For example, GOT2 is lowly expressed in hepatocellular carcinoma and is associated with poor prognosis. Inhibition of the GOT2 gene makes tumours highly dependent on glutamine metabolism for growth, rendering them exceptionally sensitive to CB‐839 [218].

The expansion of tumour cell volume relies on the outward growth of the cell membrane, a process accompanied by increased synthesis of membrane phospholipids. Targeting fatty acid synthase (FASN) and acetyl‐CoA carboxylase (ACC) can effectively intervene in this process. The FASN inhibitor TVB‐2640 has been tested in clinical trials for various cancers. In a Phase II study, combining TVB‐2640 with bevacizumab for glioblastoma treatment yielded better outcomes compared to bevacizumab monotherapy [219].

### 4.4 Targeting Ion Channels

When intracellular molecular concentrations change, cells regulate fluid flow through ion channels to balance osmotic pressure, and targeting these ion channels can inhibit cell volume changes and induce apoptosis. For example, targeting Na^+^/K^+^‐ATPase (NKA) inhibits Na^+^ efflux, causing Na^+^ accumulation inside the cell, increasing osmotic pressure and leading to cell swelling [220]. Additionally, inhibiting NKA disrupts tumour metabolism; in breast cancer, this inhibition weakens glycolysis [221]. Intracellular calcium overload due to sodium‐calcium exchanger activation suppresses β‐catenin nuclear translocation and downregulates matrix metalloproteinase (MMP) 2/9 activity, impairing tumour metastasis [222]. Increased extracellular viscosity promotes the expression of actin‐related protein 2/3 (ARP2/3), activating the Na^+^/H^+^ exchanger 1 (NHE1). Through ion exchange, intracellular osmotic pressure rises, causing cell swelling and volume expansion. This activates TRPV4, promoting calcium influx and increasing RHOA‐dependent contractility, enabling tumour cells to adapt to high‐viscosity environments for movement and migration [223]. Inhibiting chloride channels blocks chloride efflux, increasing intracellular osmotic pressure and inducing apoptosis through cell volume expansion [224]. In glioma, DCPIB inhibits tumour proliferation and migration by suppressing the JAK2/STAT3 and PI3K/Akt signalling pathways [225].

Mechanosensitive channels on cells, such as Piezo1, are activated by increased extracellular matrix stiffness or mechanical forces, promoting calcium influx and activating downstream pathways like YAP/TAZ and Rho GTPases [226]. This facilitates the expression of VEGF, CXCL16, and IGFBP2, promoting tumour angiogenesis and metastasis. In triple‐negative breast cancer, blocking the Piezo1 channel with the inhibitor GsMTx4 reduces tumour metastasis, improves the immunosuppressive microenvironment, and decreases M2 macrophage infiltration [227].

### 4.5 Impact on Cancer Immunotherapy

Research has shown that cell cycle inhibitors not only suppress tumour proliferation but also enhance tumour immunity. By targeting CDK4/6, these inhibitors suppress the Rb‐E2F axis and DNMT1 expression, enhancing tumour antigen presentation and inhibiting the proliferation of regulatory T cells (Tregs). They also activate the expression of interferon and antigen presentation‐related genes [228]. This suggests that combining CDK4/6 inhibitors with immune checkpoint inhibitors could significantly improve the efficacy of immunotherapy [229]. High expression of CDK7 is associated with poor prognosis in non‐small cell lung cancer (NSCLC). The CDK7 inhibitor THZ1 downregulates p38α and suppresses MYC transcriptional activity, reducing PD‐L1 expression and thereby promoting anti‐tumour immunity and enhancing the effectiveness of immunotherapy. Another CDK7 inhibitor, YKL‐5‐124, promotes the expression of pro‐inflammatory factors such as IFN‐γ and TNF‐α, as well as the chemokine CXCL10, facilitating T cell activation and recruitment. When combined with anti‐PD‐1 immunotherapy, YKL‐5‐124 significantly enhances immune efficacy and suppresses tumour growth [230].

### 4.6 Combined With Hormone Therapy for the Treatment of Breast Cancer

In ER+/PR+ subtype breast cancer, the activation of oestrogen receptors (ER) leads to the overactivation of Cyclin D1‐CDK4/6 and c‐Myc‐mediated cell cycle progression, promoting tumour proliferation. Combining hormone therapy with cell cycle inhibitors significantly improves treatment efficacy [231]. In first‐line treatment for advanced breast cancer, the PALOMA‐2 trial demonstrated that palbociclib combined with letrozole significantly improved outcomes in ER‐positive, HER2‐negative advanced breast cancer patients compared to letrozole alone. The median progression‐free survival (PFS) was 24.8 months versus 14.5 months (HR = 0.58), and the median overall survival (OS) was 53.9 months (49.8–60.8) versus 51.2 months (43.7–58.9) (HR = 0.956; 95% CI: 0.777–1.177; one‐sided *p* = 0.3378) [232]. In the MONARCH‐3 trial, the combination of abemaciclib with a nonsteroidal aromatase inhibitor (NSAI) significantly extended PFS compared to the placebo group, with medians of 28.2 months versus 14.8 months (HR = 0.54, 95% CI: 0.418–0.698, *p* = 0.000021) [233]. Similarly, the MONALEESA‐2 trial showed that ribociclib combined with letrozole significantly prolonged patient survival, with median OS of 63.9 months in the ribociclib group compared to 51.4 months in the placebo group (95% CI: 47.2–59.7). These findings highlight the effectiveness of combining CDK4/6 inhibitors with hormone therapy in improving outcomes for ER+/PR+ breast cancer patients [234] (Table 1).

**TABLE 1:** Combined use of cell cycle inhibitors and other antitumor drugs.

Combination regimen	Applicable cancers	Representative drug combinations	Mechanism	Clinical efficacy	Precautions	References
CDK4/6 Inhibitors + Endocrine Therapy	HR+ HER2− Breast Cancer	Palbociclib + Letrozole; Ribociclib + Fulvestrant	Cell cycle blockade (G1/S arrest) + Oestrogen signalling inhibition	**PALOMA‐2**: Median PFS 24.8 month (combinations) vs. 14.5 months (letrozole alone) **MONALEESA‐3**: Median OS 53.7 month (combinations) vs. 41.5 months (fulvestrant alone)	Monitor neutropenia; avoid strong CYP3A4 inhibitors	[235, 236]
CDK4/6 Inhibitors + PI3K/mTOR Inhibitors	HR+ Breast Cancer (PIK3CA‐mutant resistant)	Abemaciclib + Alpelisib; Ribociclib+ Everolimus	CDK4/6 inhibition+ PI3K/Akt/mTOR pathway blockade for synergistic anti‐proliferation	**SOLAR‐1**: PIK3CA‐mutant group median PFS 11.0 months (Alpelisib+ fulvestrant) vs. 5.7 months (placebo + fulvestrant) **TRINITI‐1** (Ribociclib+ everolimus): ORR 41.1%	Hyperglycemia (PI3Ki); stomatitis (mTORi)	[237, 238]
CDK4/6 Inhibitors + PARP Inhibitors	BRCA‐mutant Breast/Ovarian Cancer	Abemaciclib+ Olaparib	G1‐phase cell cycle arrest + synthetic lethality via DNA repair deficiency	**NCT03801369**: BRCA‐mutant breast cancer ORR 52%, median PFS 10.4 months	Monitor hematologic toxicity (anaemia, thrombocytopenia)	[239]
CDK4/6 Inhibitors + Immunotherapy	TNBC NSCLC	Abemaciclib+ Pembrolizumab	Enhanced tumour antigen presentation + T‐cell activation	**CheckMate 7A8**: ORR 66.7% (NIVO 480 mg Q4W + Palbo 125 mg QD + ANZ 1 mg) vs. 75% (NIVO 480 mg Q4W + Palbo 100 mg QD + ANZ 1 mg)	Immune‐related adverse events (e.g., pneumonitis, colitis)	[240]
Wee1 Inhibitors + Chemotherapy	Ovarian Cancer, SCLC	Adavosertib+ Gemcitabine/Cisplatin	G2/M checkpoint inhibition + chemotherapy‐induced DNA damage synergy	**NCT01357161**: ORR 29%, median PFS 5.5 months	Myelosuppression, diarrhoea; monitor blood counts	[241]
Pan‐CDK Inhibitors + Epigenetic Drugs	Multiple Myeloma	Dinaciclib + Panobinostat	Cell cycle/transcriptional blockade + chromatin remodelling synergy	**NCT01711528**: ORR 21%, median OS 15.3 months	Cardiac toxicity (dinaciclib); GI toxicity (panobinostat)	[242]
PLK1 Inhibitors + Chemotherapy	AML	Volasertib + Cytarabine	Mitotic blockade + chemotherapy targeting dividing cells	**NCT01957644**: CR/CRi rate 31%, median OS 8.3 months	Myelosuppression; supportive care required	[211]
Aurora Kinase Inhibitors + EGFR Inhibitors	Colorectal/Gastric Cancer	Alisertib + Cetuximab	Mitotic inhibition + EGFR signalling blockade	**Preclinical**: Synergistic tumour growth inhibition (78% TGI in models) **Early‐phase**: DCR 40%	Skin toxicity (cetuximab); neuropathy (alisertib)	[243]

### 4.7 Combination With Other Drugs

PARP is a nuclease involved in DNA repair, playing a critical role in maintaining genomic stability. In tumour cells, when DNA damage occurs, PARP1, a member of the PARP family, binds to single‐strand breaks to stabilise the structure. Inhibiting PARP prevents the formation of replication forks, exposing single‐strand DNA and exacerbating DNA damage [244]. However, due to drug resistance, PARP inhibitors like olaparib have shown limited efficacy in triple‐negative breast cancer (TNBC) patients. Combining olaparib with the CDK4/6 inhibitor palbociclib significantly suppresses the proliferation of resistant cells and enhances the tumour‐killing effects of olaparib [245] (Table 1).

The PI3K/mTOR pathway is frequently dysregulated in many cancers, leading to uncontrolled tumour proliferation. During this process, CDK4/6 expression is also upregulated. Dual inhibition of these pathways can overcome tumour resistance. For example, in the BYLieve trial, the combination of the PI3K inhibitor alpelisib with the CDK4/6 inhibitor fulvestrant in patients with PIK3CA‐mutated advanced breast cancer improved progression‐free survival (HR = 0.65, 95% CI: 0.50–0.85; one‐sided *p* < 0.001) and extended median overall survival by 7.9 months [246] (Table 1).

KRAS and EGFR mutations are prevalent in various cancers. Pancreatic ductal adenocarcinoma (PDAC) often exhibits abnormal KRAS activation and CDKN2A deletion. The loss of the tumour suppressor gene CDKN2A leads to the inactivation of the cell cycle inhibitor p16, resulting in aberrant CDK4/6 activation. Dual inhibition of these pathways provides better tumour suppression [247]. In non‐small cell lung cancer (NSCLC), EGFR mutations are a key driver, but monotherapy with EGFR tyrosine kinase inhibitors (TKIs) often leads to acquired resistance. Combining CDK4/6 inhibitors with EGFR‐TKIs like osimertinib effectively reduces resistance and improves therapeutic outcomes [248] (Table 1).

In melanoma models, combined inhibition of BRAF, MEK, and CDK4/6 significantly enhances T‐cell infiltration and anti‐tumour immunity, demonstrating potent tumour suppression. These findings highlight the potential of combination therapies targeting multiple pathways to overcome resistance and improve cancer treatment efficacy [249].

## 5 Concluding Remarks and Future Perspectives

Tumour cell size heterogeneity is a key feature of malignant progression, driven by both intrinsic genetic mutations and extrinsic microenvironmental factors. This heterogeneity reshapes tumour biology in multiple dimensions: larger cells tend to exhibit polyploidy and drug‐resistant phenotypes, while smaller cells demonstrate stem‐like properties and enhanced migratory capabilities. Cell size also influences macrophage phagocytosis, antigen presentation, and polarisation, interferes with cytotoxic granule release by T cells, and competes with immune cells for nutrients, suppressing immune cell proliferation. Targeting key regulators of cell size and combining these strategies with immunotherapy holds significant potential, although challenges such as low targeting specificity, drug resistance, and difficulties in clinical translation remain.

Future research could leverage single‐cell multi‐omics and live imaging technologies to deeply investigate the dynamic changes in cell size and their impact on proliferation, drug resistance, migration, and immune microenvironment remodelling. Innovative therapeutic strategies could include modulating the mechanical microenvironment to induce the expression of mechanosensitive genes like PIEZO1, leading to sustained activation and eventual senescence through cell volume expansion. Inhibiting cytoskeletal remodelling could hinder morphological changes, thereby preventing the invasive migration of smaller cells. Identifying size‐related biomarkers could enable precise, targeted therapy for specific patient populations. For small cells, targeted vesicle delivery of inhibitors could be employed, while for larger cells, focused ultrasound (FUS) could achieve physical ablation. Combining these approaches with multi‐target therapies, immunotherapy, and artificial intelligence‐assisted analysis to optimise treatment strategies could overcome clinical translation barriers, ultimately enabling personalised tumour treatment strategies for individual patients.

## Author Contributions

Min Zhou conceived and wrote the final manuscript. Mei Zhou and Yang Jin revised the final manuscript. All authors read and approved the final manuscript.

## Disclosure

Springer Nature remains neutral with regard to jurisdictional claims in published maps and institutional affiliations.

## Ethics Statement

The authors have nothing to report.

## Consent

The authors have nothing to report.

## Conflicts of Interest

The authors declare no conflicts of interest.

## Data Availability

The authors have nothing to report.
